# A Review of the Composition of the Essential Oils and Biological Activities of *Angelica* Species

**DOI:** 10.3390/scipharm85030033

**Published:** 2017-09-20

**Authors:** Kandhasamy Sowndhararajan, Ponnuvel Deepa, Minju Kim, Se Jin Park, Songmun Kim

**Affiliations:** 1 School of Natural Resources and Environmental Sciences, Kangwon National University, Chuncheon 24341, Korea; sowndhar1982@gmail.com (K.S.); taanishadeepa@gmail.com (P.D.); camin1121@gmail.com (M.K.); sejinpark@kangwon.ac.kr (S.J.P.); 2 Gangwon Perfume Alchemy Ltd., Co., Chuncheon 24341, Gangwon-do, Korea

**Keywords:** *Angelica*, bioactivity, essential oil, hydrodistillation, steam distillation

## Abstract

A number of *Angelica* species have been used in traditional systems of medicine to treat many ailments. Especially, essential oils (EOs) from the *Angelica* species have been used for the treatment of various health problems, including malaria, gynecological diseases, fever, anemia, and arthritis. EOs are complex mixtures of low molecular weight compounds, especially terpenoids and their oxygenated compounds. These components deliver specific fragrance and biological properties to essential oils. In this review, we summarized the chemical composition and biological activities of EOs from different species of *Angelica*. For this purpose, a literature search was carried out to obtain information about the EOs of *Angelica* species and their bioactivities from electronic databases such as PubMed, Science Direct, Wiley, Springer, ACS, Google, and other journal publications. There has been a lot of variation in the EO composition among different *Angelica* species. EOs from *Angelica* species were reported for different kinds of biological activities, such as antioxidant, anti-inflammatory, antimicrobial, immunotoxic, and insecticidal activities. The present review is an attempt to consolidate the available data for different *Angelica* species on the basis of major constituents in the EOs and their biological activities.

## 1. Introduction

In traditional systems of medicine, a number of plants have been widely used for the treatment of various disorders since ancient times. Plants are a versatile source of bioactive metabolites, including polysaccharides, phenolics, alkaloids, essential oils (EOs), steroids, lignins, resins, tannins, etc. [1]. Among them, EOs obtained from plants have various applications, especially in the health, agriculture, food, and cosmetic industries. So far, more than 3000 EOs have been isolated from about 2000 plant species, out of which 300 have been commercially used for various purposes [2]. Previous scientific studies clearly revealed that EOs possess various pharmacological properties such as antioxidant, antimicrobial, antiviral, antimutagenic, anticancer, anti-inflammatory, and immunomodulatory activities [3]. 

EOs are mainly stored in the oil ducts, resin ducts, glands, or trichomes of the plants [2]. They are a complex mixture of low molecular weight volatile compounds, mainly monoterpenes and sesquiterpenes, and their oxygenated derivatives. Each type of EO contains about 20–100 different components from a variety of chemical classes [4]. In general, the bioactivities of a particular EO are decided by its major components [3]. However, the presence of minor components also plays an essential role in the bioactivities of EOs. They can be obtained from different organs of various medicinal and aromatic plant materials using classical and advanced techniques. Hydrodistillation and steam distillation are the most important conventional techniques to isolate EOs. The gas chromatography and mass spectrometry (GC-MS) is a widely used method to determine the chemical composition of EOs [5,6]. The compositions of EOs are highly influenced by various parameters, such as harvesting season, plant organs, plant maturity, genetic diversity, nutritional status, environmental conditions, drying methods, and extraction and analysis techniques [7,8]. 

The genus *Angelica* L. belongs to the family of Apiaceae (Umbelliferae), comprises about 90 species of biennial perennial herbs that are widely distributed in Asia, Europe, and North America. In these, a total of 45 *Angelica* species (32 endemic species) are distributed in China [9]. Various species of *Angelica* have been used in the traditional systems of medicine for several centuries. Previously, several authors reported the volatile composition of different *Angelica* species using various extraction techniques such as steam distillation, hydrodistillation, solvent-free solid injector, and supercritical fluid extraction. EOs of *Angelica* species exhibit several pharmacological activities, such as antioxidant, antibacterial, antifungal, antimicrobial, and insecticidal activities. The present paper summarizes the compositions and biological activities of EOs from different *Angelica* species (Table 1 and Table 2). 

## 2. Traditional Uses of *Angelica* Species

Traditionally, *Angelica sinensis*, *Angelica gigas*, and *Angelica acutiloba* are the most important *Angelica* species, which are mainly found in Korea, China, and Japan, respectively. In China, *A. sinensis* has been used for the treatment of various ailments such as gynecological diseases, apoplexia, constipation, malaria, chills, fever, and hemorrhoids. The plant has also been used as a hematinic for nourishing blood, regulating menstruation, and relaxing bowels [10,11,12]. In the Korean traditional medicine, the root part of *A. gigas* has been to treat anemia, gynecological diseases, circulatory diseases, and arthritis. It has also been used as sedative, analgesic, and tonic agents [13,14]. *A. acutiloba* is traditionally used to treat gynecological diseases and anemia [15]. *Angelica archangelica* is commonly used in traditional medicine to cure nervousness, insomnia, stomach and intestinal disturbances, skin diseases, respiratory problems, and arthritis [16,17]. *Angelica glauca* has been used to treat bilious complaints, infantile atrophy, and constipation [18]. *Angelica dahurica* has been mainly used to treat headaches, rhinitis, toothaches, rheumatism, and sore throat [19]. *Angelica pubescentis* has been used to cure rheumatoid arthritis, headache, paralysis, and insomnia [20]. 

## 3. The Chemical Composition of Essential Oils of *Angelica* Species

**Figure 1:**
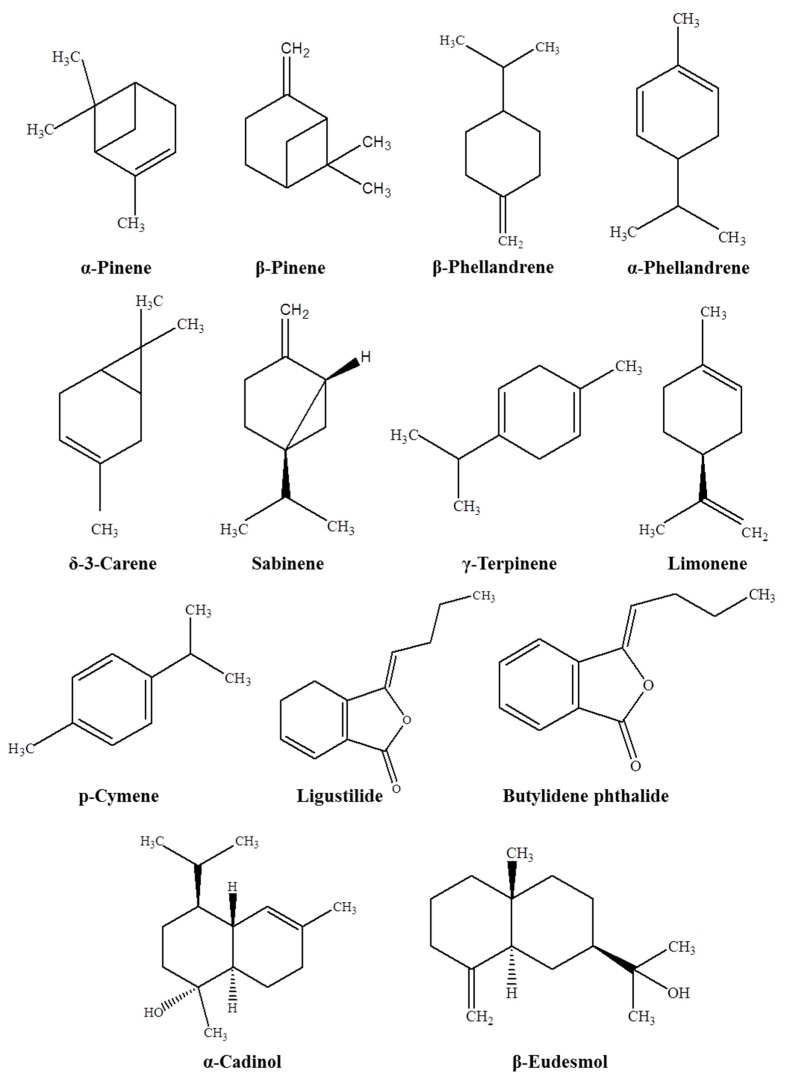
The chemical structure of some major essential oil components from *Angelica* species.

The main aim of this review is to offer an overview on the chemical composition of EOs from different species of *Angelica* growing in various countries. Table 1 shows the plant name, plant parts, extraction methods, yield, and the major components of EOs in relation to different species of *Angelica*. The published reports revealed that the EOs of the genus *Angelica* isolated by steam distillation or the hydrodistillation method mainly consist of monoterpene hydrocarbons. Figure 1 depicts the chemical structure of some of the major components of EOs from *Angelica* species.

In *A. archangelica* seed EOs, β-phellandrene (33.6–63.4%) and α-pinene (4.2–12.8%) were detected as the most abundant components [21]. On the other hand, α-pinene (21.3%), δ-3-carene (16.5%), limonene (16.4%), and α-phellandrene (8.7%) were the most abundant components in the EO of *A. archangelica* roots growing in Italy [22]. Nivinskiene et al. [23] studied the EO composition of *A. archangelica* roots collected from three habitats (Svencionys, Prienai, and Vilnius districts in Lithuania) between 1995–2002. α-Pinene (15.7–20.8%) was the major EO component in two localities, whereas β-phellandrene (13.8–18.5%) and α-pinene (11.4–15.0%) were registered as the major EO components in the third locality. The EOs contained 67.3–79.9% of monoterpenes, 9.6–19.4% of sesquiterpenes, and 3.9–6.3% of macrocyclic lactones. Chauhan et al. [17] found that the EOs of *A. archangelica* rhizomes obtained from three different altitudes of western Himalaya mainly contained dillapiole (35.93–91.55%) and nothoapiole (0.1–62.8%). Further, the authors reported that the composition of EOs varied greatly with the altitude of collection. Pasqua et al. [16] investigated the accumulation of EOs in the roots of *A. archangelica* subsp. *archangelica* at different developmental stages. A high concentration of α- and β-phellandrene was found only in taproots exceeding 5 mm in diameter.

The EO of the *A. glauca* whole plant collected from Jammu and Kashmir mainly contains α-phellandrene (18.0%), trans-carveol (16.4%), β-pinene (14.0%), β-caryophyllene (8.6%), and β-caryophyllene oxide (8.0%) [24]. Agnihotri et al. [18] investigated the composition of EO from fresh aerial parts of *A. glauca* growing in Kashmir valley in higher Himalaya (India), and found that α-phellandrene (13.5%), *trans*-carveol (12.0%), and β-pinene (11.7%) were the major components. The EOs from the roots of *A. glauca* collected from two alpine Himalayan locations in Uttarakhand (India) highly contain *(Z)*-ligustilide (40.6–53.0%) and *(Z)*-butylidene phthalide (20.7–32.8%) [25].

Kim et al. [29] determined the EO composition from the rhizomes of *A. gigas*, *A. sinensis,* and *A. acutiloba* by solvent-free solid injector method. Coumarin derivatives such as decursinol angelate (16.83%) and decursin (29.34%) were found to be the most abundant components, followed by lomatin (10.25%), and marmesin (9.33%) in *A. gigas*. Butylidene dihydro-phthalide, (15.23%), butylidene phthalide (14.27%), furfural (16%), and camphene (10.66%) were the main components in *A. sinensis*. Similarly, butylidene phthalide (17.82%) and furfural (13.67%) were registered as the major components in *A. acutiloba*. 

Sowndhararajan et al. [14] compared the EO composition of *A. gigas* root by steam distillation and supercritical carbon dioxide extract (SC-CO_2_). The EO mainly composed of monoterpene hydrocarbons (52.83%), followed by oxygenated sesquiterpenes (25.53%). In these, α-pinene (28.64%), β-eudesmol (14.80%), nonane (8.49%), and γ-eudesmol (5.97%) were the major components in the EO of *A. gigas* root. However, decursin (40.13%) and decursinol angelate (28.44%) were detected as the most abundant components in SC-CO_2_. α-Pinene (30.89%) was also the major component in the EO of *A. gigas* extracted by simultaneous steam distillation and extraction method [31]. In another study, the roots of *A. gigas* and *A. acutiloba* were collected from the field of Snyder Research and Extension Farm Rutgers University, New Jersey, and analyzed for their EO composition. The main constituents of the *A. gigas* root EO were ligustilide (47%) and γ-terpinene (14%). In the case of *A. acutiloba* root EO, α-pinene (32%) and nonane (25%) were the major components [28].

Chen et al. [30] compared the volatile compositions of *A. acutiloba* roots, stems, and leaves using steam distillation and headspace solid-phase microextraction (HS-SPME). In all three parts, a total of 61 and 33 compounds were detected by SD and HS-SPME, respectively. In the steam distillation, 3n-butyl phthalide, γ-terpinene, *p*-cymene, and *cis*-β-ocimene were the main compounds. On the other hand, γ-terpinene and *p*-cymene were the main compounds in HS-SPME. Further, the authors reported that monoterpene components were found to be higher in the HS-SPME sampling method when compared with steam distillation.

In the EO of *A. major*, α-pinene (21.8%) and *cis*-β-ocimene (30.4%) were found to be the most abundant components [55]. The main components in *A. dahurica* EO were α-pinene (46.3%), sabinene (9.3%), myrcene (5.5%), 1-dodecanol (5.2%), and terpinen-4-ol (4.9%). In regards to *A. pubescentis* root EO, α-pinene (37.6%), *p*-cymene (11.6%), limonene (8.7%), and cryptone (6.7%) were found to be the major components [20]. Champakaew et al. (2015) found that 3-*N*-butylphthalide, butylidene phthalide, ligustilide, and di-iso-octyl phthalate were the main components in *A. sinensis* EO. The composition of EOs of the stem and leaves of *Angelica urumiensis* were studied by Mohammadi et al. [38]. In the EO from the leaves, α-cadinol (20.2%), hexahydrofarnesyl acetone (10.03%), and 1-dodecanol (7.55%) were the major components. On the other hand, α-cadinol (9.24%) and δ-cadenine (6.11%) were the major components in the EO from the stem. The EO compositions of *A. pancicii* were compared by GC-MS liquid injection and headspace-GC-MS modes. In total, 40 compounds were identified in the EO by GC-MS liquid injection, and 44 by HS-GC-MS. In both cases, the main components were β-phellandrene, α-pinene, and α-phellandrene [37]. Caryophyllene oxide (61.7%) and α-pinene (67.2%) were detected as the most abundant components in EOs of *A. viridiflora* and *A. cincta* aerial parts, respectively [39].

## 4. Biological Activities of *Angelica* Essential Oils

### 4.1. Antioxidant

1,1-Diphenyl-2-picrylhydrazil (DPPH) and 2,2-azino-bis(3ethylbenzo-thiazoline-6-sulfonic acid (ABTS) radical scavenging activities are extensively used measures to evaluate the antioxidant potential of plant extracts or compounds. DPPH, nitrite inhibition, and reducing power were determined to assess the antioxidant activity of *Angelica koreana* EO and its major components. *m*-Cresol (56.12%) showed stronger DPPH scavenging activity than EO (19.31%) and sabinene (4.45%) at the concentration of 16 mg/mL. Additionally, sabinene exhibited the strongest reducing power and nitric oxide scavenging activities than the EO fraction or *m*-cresol [36]. Irshad et al. [24] reported that *A. glauca* EO exhibited good DPPH radical scavenging and peroxidation inhibition activities. *Angelica* seed oil showed 39% of DPPH radical scavenging activity at the concentration of 200 μg/mL [40]. The antioxidant activity of *A. sinensis* was investigated by DPPH, ABTS, and beta-carotene bleaching assays. *A. sinensis* EO and coniferyl ferulate rich fractions 1 and 2 showed strong DPPH (IC_50_ of 194.7, 42.4 and 15.2 μg/mL, respectively) and ABTS (IC_50_ of 98.8, 15.9 and 7.8 μg/mL, respectively) radical scavenging activities. Further, coniferyl ferulate rich fractions 1 and 2 exhibited good β-carotene bleaching activity with IC_50_ values of 11.0 and 2.0 μg/mL, respectively [46]. In another study, the DPPH radical scavenging activity of *A. archangelica* EO, α-terpineol, phenyl ethyl alcohol, and their combination were determined. The IC_50_ values of *A. archangelica* EO, α-terpineol, and their EO-based combination were 1.04, 66.6, and 3.89 μL/mL, respectively [42].

### 4.2. Antimicrobial

*A. koreana* EO and its main components, sabinene and *m*-cresol, showed antifungal activity against different species of *Aspergillus* and *Trichophyton* with minimal inhibitory concentrations (MICs) of 125–1000 μg/mL. In addition, EO exhibited synergistic activity when combined with itraconazole [36]. The EO of *A. glauca* showed appreciable antimicrobial activity against selected strains of bacteria (*Staphylococcus aureus*, *Bacillus subtilis*, *Escherichia coli,* and *Pasteurella multocida*) and fungi (*Candida albicans*, *Microsporum canis*, *Aspergillus flavus,* and *Fusarium solani*). Among the bacterial strains tested, *Escherichia coli* and *Staphylococcus aureus* were the most sensitive bacteria with minimum inhibitory concentration (MIC) values of 141.3 and 159.3 µg/mL, respectively. In regards to fungal strains, *Microsporum canis* was the most sensitive organism with a MIC value of 178.1 µg/mL [24].

The EO of *A. archangelica* root showed considerable antimicrobial activity against *Clostridium difficile, Clostridium perfringens*, *Enterococcus faecalis*, *Eubacterium limosum*, *Peptostreptococcus anaerobius*, and *Candida albicans*. Further, *A. archangelica* EO showed a weaker antimicrobial activity against the intestinal microflora such as bifidobacteria and lactobacilli. In another study, the EO showed antifungal activity against some species of the Fusarium genus, *Botrytis cinerea*, and *Alternaria solani* [22,27]. A combination of *A. archangelica* EO: Phenyl ethyl alcohol (PEA): α-terpineol (1:1:1) inhibited the growth of *Aspergillus flavus* NKDW-7 (aflatoxigenic strain) and aflatoxin B1 production at 2.25 and 2.0 μL/mL, respectively. At the concentration of 2.0 μL/mL, the combination showed a >90% decrease in ergosterol content in the plasma membrane of *Aspergillus flavus* [42]. 

Cavaleiro et al. [55] evaluated the antifungal activity of the EO of *Angelica major* and its major components, α-pinene and *cis*-β-ocimene, against clinically important yeasts and molds. *A. major* EO exhibited a broad spectrum of antifungal activity, including all tested fungi (animal and human pathogenic species or spoilage fungi): *Candida* spp., *C. neoformans*, *Aspergillus* spp., and dermatophytes. α-Pinene was more active against all of the tested fungi than *cis*-β-ocimene. *A. sinensis* and *A. dahurica* EOs exhibited significant antibacterial activity against three mastitis-causing pathogens: *Staphylococcus aureus*, *Staphylococcus chromogenes*, and *Streptococcus uberis* [47]. Tabanca et al. [20] reported that *A. pubescentis* root EO exhibited weak antifungal activity against *Colletotrichum acutatum*, *Colletotrichum fragariae*, and *Colletotrichum gloeosporioides*. In the case of *A. dahurica* root EO, there was no antifungal activity observed against tested fungal strains. 

### 4.3. Insecticidal

EOs from the root of *A. dahurica* and *A. pubescentis* were studied as pest management prospectives. When compared with *A. pubescentis* EO, *A. dahurica* EO showed better biting deterrent and insecticidal activity against *Aedes aegypti* and *Stephanitis pyrioides*. In mosquito bioassays, components of *A. dahurica* EO, 1-dodecanol and 1-tridecanol, showed antibiting deterrent activity against *Aedes aegypti* [20]. Chung et al. [44] investigated the immunotoxicity effect of EOs from the leaves of *A. anomala*, *A. cartilagino-marginata* var. *distans*, *A. czernevia*, *A. dahurica*, *A. decursiva*, *Angelica fallax*, *A. gigas,* and *A. japonica*. Among them, the EO of *A. dahurica* showed a significant toxic effect against early fourth-stage larvae of *Aedes aegypti,* with a LC_50_ value of 43.12 ppm. In another study, out of 33 plant species tested, *A. sinensis* EO showed the best repellent activity against *Aedes aegypti,* with a median complete protection time of 7.0 h [35].

### 4.4. Behavioral

Repeated administration of nicotine can produce behavioral sensitization, and this is a good model for studying drug addiction. Zhao et al. [43] reported that the inhalation of *A. gigas* EO significantly ameliorated nicotine-induced behavioral sensitization by decreasing dopamine release in the nucleus accumbens and locomotor activity in repeated nicotine-induced rats. Pathak et al. [41] found that the EO of the *A. archangelica* root exhibited antiseizure activity against electrically and chemically-induced seizures in mice. Chen et al. [50] investigated the anxiolytic activity of *Angelica* EO in a mice model. The results revealed that the EO of *Angelica* exhibited considerable anxiolytic-like effects at the concentration of 30.0 mg/kg (orally), as measured in the elevated plus-maze, the light/dark, and the stress-induced hyperthermia tests. In addition, *Angelica* EO significantly improved the behavioral performances in the social interaction test of anxiety and the hole-board test of exploration and locomotor activity in rats [51]. Sharma et al. [45] reported that the EO of *A. glauca* exhibited broncho-relaxant activity against histamine and ovalbumin-induced bronchoconstriction in guinea pigs by decreasing absolute blood eosinophil count, serum levels of immunoglobulin E, and the number of eosinophils and neutrophils in bronchoalveolar lavage fluid. Sowndhararajan et al. [14] investigated the effect of inhalation of EO of *A. gigas* root on electroencephalographic activity in humans. The results revealed that absolute low beta significantly increased at left temporal and left parietal region during the inhalation of the EO of *A. gigas* root, and these changes may contribute to the enhancement of language learning abilities in humans.

### 4.5. Anti-Inflammatory

Zhang et al. [32] used the metabonomics based on GC-MS to study the possible anti-inflammatory mechanisms of EO of *A. sinensis* in rats with acute inflammation. In the carrageenan-injected rats, treatment with the EO of *A. sinensis* significantly restored the levels of prostaglandin E2, histamine, and 5-hydroxytryptamine in the inflammatory fluid, similar to the normal group. GC-MS analysis identified 14 metabolite biomarkers detected in the inflammatory fluid. Zhong et al. [48] evaluated the anti-inflammatory effect of EOs obtained from processed products of *A. sinensis*. For this purpose, EOs from stir-fried *A. sinensis*, fried *A. sinensis* with alcohol, cooked *A. sinensis* with soil, and fried *A. sinensis* with sesame oil were applied to intervene the carrageenan-induced acute inflammation of the model rats. The results showed that the EOs of *A. sinensis* significantly inhibited the release of prostaglandin E2, histamine, 5-hydroxytryptamine, and tumor necrosis factor-α. Furthermore, *A. sinensis* exhibited an anti-inflammatory effect against the lipopolysaccharide (LPS)-induced inflammation rat model by regulating the Krebs cycle, enhancing the glucose content, and restoring the fatty acid metabolism [33]. 

Li et al. [49] investigated the effects of *A. sinensis* EO on the LPS-induced acute inflammation rat model. *A. sinensis* EO exhibited anti-inflammatory and liver protection effects by inhibiting the secretion of the pro-inflammatory cytokines (tumor necrosis factor-α, interleukin-1β, and interleukin-6), the inflammatory mediators (histamine, 5-hydroxytryptamine, prostaglandin E2, and nitric oxide), the inflammation-related enzymes (inducible nitric oxide synthase and cyclooxygenase 2), as well as promoting the production of the anti-inflammatory cytokines interleukin-10. Wang et al. [19] reported that the EO of *A. dahurica* (at 100 mg/kg) showed anti-inflammatory activity against xylene-induced ear swelling and carrageenan-induced paw edema in a mice model. In addition, the EO significantly alleviated Freund’s complete adjuvant-induced arthritis in rats by improving hind paw swelling and reducing the serum levels of nitric oxide, tumor necrosis factor-α, prostaglandin E2, and serum nitric oxide synthase activity. 

### 4.6. Skin Permeation Enhancer

It is well known that EOs can reversibly overcome the stratum corneum barrier to improve the skin permeation of drugs. Chen et al. [53] studied the penetration enhancement effect of five EOs (clove, *Angelica*, Chuanxiong, *Cyperus*, and cinnamon) on the transdermal drug delivery of ibuprofen using dysmenorrheal model mice. Among five EOs tested, Chuanxiong and *Angelica* oils effectively enhanced the transdermal drug delivery of ibuprofen. In another study, turpentine, *Angelica*, Chuanxiong, *Cyperus*, cinnamon, and clove oils (at 3% *w*/*v*) were evaluated for the potential to enhance the skin penetration of ibuprofen in rats. When compared with azone, the tested EOs had significantly higher penetration enhancement effect and lower skin irritation potential. The results revealed that EOs can enhance the skin permeation of ibuprofen mainly by disturbing the stratum corneum lipids [54].

## 5. Conclusions

EOs have been isolated from different plant parts of *Angelica* species. The most abundant components in the EOs were α-pinene, β-pinene, α-phellandrene, β-phellandrene, δ-3-carene, sabinene, γ-terpinene, limonene, *p*-cymene, ligustilide, butylidene phthalide, α-cadinol, and β-eudesmol. Based on the previous reports, the EOs from different *Angelica* species exhibit appreciable antioxidant, antimicrobial, insecticidal and anti-inflammatory activities. In addition, EOs significantly enhance behavioral performances and promote the skin permeation of drugs. Among the different *Angelica* species, *A. archangelica*, *A. sinensis,* and *A. dahurica* were the most studied plant species in relation to the biological activities of EOs. This review will offer a scientific basis for future studies in relation to biological activities of EO-bearing plants.

## Figures and Tables

**Table 1:** The isolation of essential oils and extracts from different *Angelica* species, and their major components.

S. No.	Species	Parts	Extraction Method; Extraction Time; Yield	Place of Collection	Major Components	References
1	***Angelica archangelica* L.**	Seeds (fruits) from three habitats	Hydrodistillation; 2 h; 0.8–1.4%	Svencionys, Prienai and Vilnius districts in Lithunia	β-phellandrene (33.6–63.4%) and α-pinene (4.2–12.8%)	[21]
		Fruit of two chemotypes	Steam distillation; 5 h; 0.17–0.51%	Reykjavik, Iceland	α-pinene (41.4%, 28.9%, 14.4%), bicyclogermacrene (10.1%), and β-phellandrene (37.8% and 55.2%)	[26]
		Root (1–2, 3–4 and >5 mm)	Hydrodistillation; 30 min	Rome, Italy	α-pinene (23.89–32.69%) and δ-3-carene (3.41–17.07%)	[16]
		Root (3 habitats)	Hydrodistillation; 2 h; 0.2–0.5%	Svencionys, Prienai and Vilnius districts in Lithuania	α-pinene (15.7–20.8%), δ-3-carene (15.4–16.9%), limonene (8.0–9.2%), β-phellandrene (13.5–15.4%), α-phellandrene (8.0–9.1%), and *p*-cymene (6.8–10.6%)	[23]
		Root (3 different altitudes)	Hydrodistillation; 3 h; 0.28–0.35%	Uttarakashi, Rudraprayag and Pothiwasa in Uttarakhand, India	dillapiole (35.93–91.55%) and nothoapiole (0.14–62.81%)	[17]
		Root	Hydrodistillation; 2 h; 0.9%	Urbino, Italy	α-pinene (21.3%), δ-3-carene (16.5%), limonene (16.4%), and α-phellandrene (8.7%)	[22,27]
2	***Angelica acutiloba*** **(Siebold & Zucc.) Kitag.**	Leaves, petiole and root	Hydrodistillation; 3 h; 0.44%	Rutgers University, New Brunswick, NJ, USA	Leaves: ligustilide (11.61%) and butylidene phthalide (7.29%) Petiole: butylidene phthalide (10.76%) Root: nonane (24.85%) and α-pinene (31.59%)	[28]
		Root	Solvent free solid injector; injection time—5 min and pre-heating time—7 min)	Yeosu Province, Republic of Korea	butylidene phthalide (17.82%), furfural (13.67%), 2-furanmethanol (11.97%), 5-methyl furfural (8.50%), maltol (7.28%), and butylidene dihydro-phthalide (5.78%)	[29]
		Root, stem and leaves	Steam distillation; 5 h; 0.05 (root), 0.06 (stem), and 0.12 (leaves)	Nantou, Taiwan	3n-butyl phthalide (30.8–37.9%), γ-terpinene (21.1–27.2%), *p*-cymene (3.6–11.6%), and *cis*-β-ocimene (7.0–7.4%)	[30]
			Headspace-solid phase microextraction; 20 min	Nantou, Taiwan	γ-terpinene (41.2–52.1%), *p*-cymene (10.6–17.0%), β-myrcene (6.7–8.6%), *cis*-β-ocimene (4.9–7.4%), and alloocimene (4.2–5.3%)	[30]
3	***Angelica glauca* Edgew**	Whole plant	Hydrodistillation; 3 h, 0.17%	Jammu and Kashmir, Pakistan	α-phellandrene (18.0%), β-pinene (14.0%), *trans*-carveol (16.4%), β-caryophyllene (8.6%), and β-caryophyllene oxide (8.0%).	[24]
		Aerial parts	Hydrodistillation; 3 h; 0.12%	Khillanmarg areas of Kashmir, India	α-phellandrene (13.5%), *trans*-carveol (12.0%), β-pinene (11.7%), thujene (7.5%), β-caryophyllene oxide (7.2%), β-caryophyllene (7.0%), γ-terpinene (6.7%), nerolidol (6.5%), and β-bisabolene (5.2%)	[18]
		Root	Hydrodistillation; 5 h; 0.3% and 1.8%	Himalayan locations of Uttarakhand, India	*(Z)*-ligustilide (40.6–53.0%), *(Z)*-butylidene phthalide (20.7–32.8%), and *(E)*-butylidene phthalide (2.5–5.9%)	[25]
4	***Angelica gigas* Nakai**	Leaves, petiole and root	Hydrodistillation; 3 h; 0.18%	Rutgers University, New Brunswick, NJ, USA	Leaves: nonane (10.75%), α-pinene (33.07%), and germacrene (10.05%) Petiole: nonane (8.85%), α-pinene (40.59%), β-phellandrene (7.52%), and myrcene (6.38%) Root: γ-terpinene (14.08%) and ligustilide (46.63%)	[28]
		Root	Hydrodistillation; 4 h	Yeosu Province, Republic of Korea	nonane (19.99%), α-pinene (44.31%), camphene (6.66%), and δ-limonene (6.26%)	[29]
			Solvent-free solid injector; injection time—5 min and pre-heating time—7 min)	Yeosu Province, Republic of Korea	decursin (29.34%), decursinol angelate (16.83%), lomatin (10.25%), and marmesin (9.33%)	[29]
			Simultaneous steam distillation (n-pentane/diethyl ether); 2 h; 0.31%	Gwangju, Republic of Korea	α-pinene (30.89%), 2,4,6-trimethyl heptane (13.39%), α-limonene (4.29%), and camphene (4.10%)	[31]
			Steam distillation; 1 h 30 min; 0.31%	Pyeongchang, Republic of Korea	α-pinene (28.64%), β-eudesmol (14.80%), nonane (8.49%), and γ-eudesmol (5.97%)	[14]
			Supercritical CO_2_ extraction; 1 h; 1.67%	Pyeongchang, Republic of Korea	decursin (40.13%), decursinol angelate (28.44%), and β-eudesmol (7.84%)	[14]
5	***Angelica sinensis* (Oliv.) Diels**	Root	Hydrodistillation; 8 h; 0.3%	Gansu Province, China	*(Z)*-ligustilide 78.61% and *(Z)*-butylidenephthalide 7.99%	[32,33,34]
			Solvent free solid injector; injection time—5 min and pre-heating time—7 min)	Yeosu Province, Republic of Korea	butylidene dihydro-phthalide, (15.23%), butylidene phthalide (14.27%), furfural (16%), camphene (10.66%), and 4-pyridinol (7.17%)	[29]
			Steam distillation; 3 h; 0.02%	Chiang Mai province, Thailand	3-*N*-butylphthalide, butylidenephthalide, ligustilide and di-iso-octyl phthalate	[35]
6	***Angelica koreana* Maxim.**	Root	Steam distillation; 0.28%	Jinbu, Gangwon-do, Republic of Korea	sabinene (31.85%), m-cresol (4.46%), α-pinene (4.00%), and α-bisabolol (3.63%)	[36]
7	***Angelica dahurica*** **(Fisch. Ex Hoffm.) Benth. & Hook.**	Root	Supercritical CO_2_ extraction; 2 h; 1.8%	Jilin, China	dodecyl alcohol (13.71%), elemene (7.54%), hexadecanoic acid, ethyl ester (7.32%), 1-pentadecanol (6.08%), and α-pinene (6.25%),	[19]
			Hydrodistillation; 3 h; 0.45%	Beijing, China	α-pinene (46.3%), sabinene (9.3%), myrcene (5.5%), 1-dodecanol (5.2%), and terpinen-4-ol (4.9%).	[20]
8	***Angelica pancicii*** **Vandas ex Velen.**	Root	Hydrodistillation; 2 h	Balkan mountains, Serbia	Liquid and headspace injection modes: β-phellandrene (54.9% and 60.1%), α-pinene (14.5% and 20.1%), and α-phellandrene (4.5% and 4.3%).	[37]
9	***Angelica pubescentis* Maxim.**	Root	Hydrodistillation; 3 h; 0.65%	Beijing, China	α-pinene (37.6%), *p*-cymene (11.6%), limonene (8.7%), and cryptone (6.7%)	[20]
10	***Angelica urumiensis* (Mozaffarian)**	Stem	Hydrodistillation; 3 h; 0.2%	Uremia, Province West Azerbaijan, Iran	Stem: α-cadinol (9.24%), (epi)-α-cadinol (5.76%), and δ-cadenine (6.11%)	[38]
11	***Angelica urumiensis* (Mozaffarian)**	Leaves	Hydrodistillation; 3 h; 0.18%	Uremia, Province West Azerbaijan, Iran	Leaves: α-cadinol (20.2%), hexahydrofarnesyl acetone (10.03%), 1-dodecanol (7.55%), linoleic acid (6.37%) and oleic acid (5.34%)	[38]
12	***Angelica viridiflora*** **(Turcz.) Benth. ex Maxim.**	Aerial parts	Steam distillation; 2 h; 0.2%	Shkotovskii District, Primorsky Krai, Russia	caryophyllene oxide (61.7%) and 3,4-dimethyl-3-cyclohexan-1-carboxaldehdye (5.8%)	[39]
13	***Angelica cincta* Boissieu**	Aerial parts	Steam distillation; 2 h; 0.2%	Shkotovskii District, Primorsky Krai, Russia	α-pinene (67.2%), sabinene (5.8%) and β-pinene (4.9%)	[39]

**Table 2:** Biological activities of essential oils from different *Angelica* species.

S. No.	Species	Parts	Biological activity	Model	References
1	***Angelica archangelica* L.**	Seeds	Antioxidant	Aldehyde/Carboxylic Acid Assay, DPPH radical scavenging assay, and Malonaldehyde/Gas Chromatography Assay	[40]
		Fruit of two chemotypes	Cytotoxic effect	Human pancreas cancer cell line PANC-1 and the mouse breast cancer cell line Crl	[26]
		Root	Anti-seizure	Maximal electroshock and pentylenetetrazol-induced seizures in mice	[41]
			Anti-aflatoxigenic and antioxidant activities	*Aspergillus flavus* DPPH radical scavenging assay	[42]
			Antimicrobial	*Fusarium* genus, *Botrytis cinerea*, *and Alternaria solani*, *Clostridium difficile*, *Clostridium perfringens*, *Enterococcus faecalis*, *Eubacterium limosum*, *Peptostreptococcus anaerobius*, and *Candida albicans*	[22,27]
2	***Angelica gigas* Nakai**	Root	Nicotine Sensitization	Repeated nicotine-induced locomotor activity and extracellular dopamine levels in the nucleus accumbens of rats	[43]
			Human EEG	Increased absolute low beta (left temporal and left parietal) activity	[14]
		Leaves	Immunotoxicity	Larvae of *Aedes aegypti*	[44]
3	***Angelica glauca* Edgew**	Whole plant	Antioxidant, antimicrobial, and phytotoxic	Bacteria: *Staphylococcus aureus*, *Bacillus subtilis*, *Escherichia coli*, and *Pasteurella multocida* Fungi: *Candida albicans*, *Microsporum canis*, *Aspergillus flavus* and *Fusarium solani*. DPPH radical scavenging assay Phytotoxic activity against *Lemna minor*	[24]
			Broncho-relaxant	Airway was induced using histamine aerosol in guinea pigs and ovalbumin aerosol in albino mice.	[45]
4	***Angelica sinensis* (Oliv.) Diels**	Root	Anti-inflammatory	Carrageenan-induced rats	[32]
			Antioxidant	DPPH, ABTS scavenging, and β-carotene bleaching assays.	[46]
			Anti-inflammatory	Carrageenan-induced rats and mechanism by plasma metabolomics approach	[34]
			Antibacterial	*Staphylococcus aureus*, *Staphylococcus chromogenes*, and *Streptococcus uberis*	[47]
			Anti-inflammatory	Carrageenan-induced acute inflammation model rats	[48]
			Anti-inflammatory	Lipopolysaccharide-induced inflammation rat model	[33,49]
			Anxiolytic	Elevated plus-maze, light/dark and stress-induced hyperthermia tests	[50]
				Social interaction test of anxiety and the hole-board test	[51]
			Repellent	Against *Aedes aegypti*	[35]
5	***Angelica koreana* Maxim.**	Root	Antifungal and antioxidant	*Aspergillus* (*A. flavus*, *A. fumigaus*, *A. niger*, *A. terreus* and *A. versicolor*) and *Trichophyton* (*T. mentagrophytes*, *T. rubrum* and *T. tonsurans*) species DPPH scavenging, nitrite inhibition, and reducing power	[36]
6	***Angelica dahurica*** **(Fisch. Ex Hoffm.) Benth. & Hook.**	Root	Anti-inflammatory and immunomodulating properties	Xylene-induced acute ear swelling and carrageenan-induced acute paw edema in mice; anti-inflammatory and immunomodulating properties in Freund’s complete adjuvant (FCA)-induced arthritis in rats.	[19]
			Enhance sensitivity of MCF-7/ADR breast cancer cells to doxorubicin	MDR human breast cancer MCF-7/ADR cells	[52]
			Insecticidal	Yellow fever mosquito, *Aedes aegypti*, and azalea lace bugs, *Stephanitis pyrioides*	[20]
			Antibacterial	*Staphylococcus aureus*, *Staphylococcus chromogenes*, and *Streptococcus uberis*	[47]
			Immunotoxicity	Larvae of *Aedes aegypti*	[44]
7	***Angelica pubescentis* Maxim.**	Root	Antifungal and Insecticidal	*Colletotrichum acutatum*, *Colletotrichum fragariae*, and *Colletotrichum gloeosporioides*, Yellow fever mosquito, *Aedes aegypti*, and azalea lace bugs, *Stephanitis pyrioides*	[20]
8	***Angelica anomala*** **Avé-Lall., *Angelica cartilagino-marginata* var. *distans*, *Angelica czernevia*, *Angelica decursiva* (Miq.) Franch. & Sav., *Angelica fallax* H. Boissieu , *Angelica japonica* A. Gray**	Leaves	Immunotoxicity	Larvae of *Aedes aegypti*	[44]
9	***Angelica* species**	Root	Penetration Enhancers for Transdermal Administration of Ibuprofen	Therapeutic efficacy of ibuprofen with essential oil was evaluated using dysmenorrheal model mice	[53]
			Skin permeation of drugs	Skin permeation of ibuprofen across rat abdominal skin	[54]

DPPH: 1,1-diphenyl-1-picrylhydrazyl; ABTS: 2,2-azino-bis(3ethylbenzo-thiazoline-6-sulfonic acid); EEG: electroencephalographic activity; MDR: multidrug resistance.
